# Supplementary material for: HIVprotI: an integrated web based platform for prediction and design of HIV proteins inhibitors
Source: J Cheminform. 2018 Mar 9;10:12. doi: 10.1186/s13321-018-0266-y (PMC5845081; doi:10.1186/s13321-018-0266-y)
Supplement: Supplementary file 2 — Additional file 2. Source code of HIVProtI web server. [file 13321_2018_266_MOESM2_ESM.zip › HIVprotI_Source-code/hivprotI/weka-3-6-6/doc/weka/classifiers/trees/RandomTree.html]

RandomTree


---


|  |  |  |
| --- | --- | --- |
| **Overview** \| **Package** \| **Class** \| **Tree** \| **Deprecated** \| **Index** \| **Help** \| **Weka's home** | |  |
| **PREV CLASS**   **NEXT CLASS** | **FRAMES**    **NO FRAMES**     **All Classes** |  |
| SUMMARY: NESTED \| FIELD \| CONSTR \| METHOD | DETAIL: FIELD \| CONSTR \| METHOD |  |


---


## weka.classifiers.trees Class RandomTree

```
java.lang.Object
  weka.classifiers.Classifier
      weka.classifiers.trees.RandomTree
```

**All Implemented Interfaces:**: java.io.Serializable, java.lang.Cloneable, CapabilitiesHandler, Drawable, OptionHandler, Randomizable, RevisionHandler, WeightedInstancesHandler

---

``` public class RandomTree extends Classifier implements OptionHandler, WeightedInstancesHandler, Randomizable, Drawable ```

Class for constructing a tree that considers K randomly chosen attributes at each node. Performs no pruning. Also has an option to allow estimation of class probabilities based on a hold-out set (backfitting).


Valid options are:

```
 -K <number of attributes>
  Number of attributes to randomly investigate
  (<0 = int(log_2(#attributes)+1)).
```

```
 -M <minimum number of instances>
  Set minimum number of instances per leaf.
```

```
 -S <num>
  Seed for random number generator.
  (default 1)
```

```
 -depth <num>
  The maximum depth of the tree, 0 for unlimited.
  (default 0)
```

```
 -N <num>
  Number of folds for backfitting (default 0, no backfitting).
```

```
 -U
  Allow unclassified instances.
```

```
 -D
  If set, classifier is run in debug mode and
  may output additional info to the console
```

**Version:**
:   $Revision: 5535 $

**Author:**
:   Eibe Frank (eibe@cs.waikato.ac.nz), Richard Kirkby (rkirkby@cs.waikato.ac.nz)

**See Also:**: Serialized Form

---

**Field Summary**

**Fields inherited from interface weka.core.Drawable**

`BayesNet, Newick, NOT_DRAWABLE, TREE`


**Constructor Summary**

`RandomTree()`


| **Method Summary** | |
| --- | --- |
| `java.lang.String` | `allowUnclassifiedInstancesTipText()`             Returns the tip text for this property |
| `void` | `backfitData(Instances data)`             Backfits the given data into the tree. |
| `void` | `buildClassifier(Instances data)`             Builds classifier. |
| `double[]` | `distributionForInstance(Instance instance)`             Computes class distribution of an instance using the decision tree. |
| `boolean` | `getAllowUnclassifiedInstances()`             Get the value of NumFolds. |
| `Capabilities` | `getCapabilities()`             Returns default capabilities of the classifier. |
| `int` | `getKValue()`             Get the value of K. |
| `int` | `getMaxDepth()`             Get the maximum depth of trh tree, 0 for unlimited. |
| `double` | `getMinNum()`             Get the value of MinNum. |
| `int` | `getNumFolds()`             Get the value of NumFolds. |
| `java.lang.String[]` | `getOptions()`             Gets options from this classifier. |
| `java.lang.String` | `getRevision()`             Returns the revision string. |
| `int` | `getSeed()`             Gets the seed for the random number generations |
| `java.lang.String` | `globalInfo()`             Returns a string describing classifier |
| `java.lang.String` | `graph()`             Returns graph describing the tree. |
| `int` | `graphType()`             Returns the type of graph this classifier represents. |
| `java.lang.String` | `KValueTipText()`             Returns the tip text for this property |
| `java.util.Enumeration` | `listOptions()`             Lists the command-line options for this classifier. |
| `static void` | `main(java.lang.String[] argv)`             Main method for this class. |
| `java.lang.String` | `maxDepthTipText()`             Returns the tip text for this property |
| `java.lang.String` | `minNumTipText()`             Returns the tip text for this property |
| `java.lang.String` | `numFoldsTipText()`             Returns the tip text for this property |
| `int` | `numNodes()`             Computes size of the tree. |
| `java.lang.String` | `seedTipText()`             Returns the tip text for this property |
| `void` | `setAllowUnclassifiedInstances(boolean newAllowUnclassifiedInstances)`             Set the value of AllowUnclassifiedInstances. |
| `void` | `setKValue(int k)`             Set the value of K. |
| `void` | `setMaxDepth(int value)`             Set the maximum depth of the tree, 0 for unlimited. |
| `void` | `setMinNum(double newMinNum)`             Set the value of MinNum. |
| `void` | `setNumFolds(int newNumFolds)`             Set the value of NumFolds. |
| `void` | `setOptions(java.lang.String[] options)`             Parses a given list of options. |
| `void` | `setSeed(int seed)`             Set the seed for random number generation. |
| `java.lang.String` | `toGraph()`             Outputs the decision tree as a graph |
| `int` | `toGraph(java.lang.StringBuffer text, int num)`             Outputs one node for graph. |
| `java.lang.String` | `toString()`             Outputs the decision tree. |

**Methods inherited from class weka.classifiers.Classifier**

`classifyInstance, debugTipText, forName, getDebug, makeCopies, makeCopy, setDebug`

**Methods inherited from class java.lang.Object**

`equals, getClass, hashCode, notify, notifyAll, wait, wait, wait`

**Constructor Detail**

### RandomTree

```
public RandomTree()
```


**Method Detail**

### globalInfo

```
public java.lang.String globalInfo()
```

:   Returns a string describing classifier

    :   **Returns:**: a description suitable for displaying in the explorer/experimenter gui

---


### minNumTipText

```
public java.lang.String minNumTipText()
```

:   Returns the tip text for this property

    :   **Returns:**: tip text for this property suitable for displaying in the explorer/experimenter gui

---


### getMinNum

```
public double getMinNum()
```

:   Get the value of MinNum.

    :   **Returns:**: Value of MinNum.

---


### setMinNum

```
public void setMinNum(double newMinNum)
```

:   Set the value of MinNum.

    :   **Parameters:**: `newMinNum` - Value to assign to MinNum.

---


### KValueTipText

```
public java.lang.String KValueTipText()
```

:   Returns the tip text for this property

    :   **Returns:**: tip text for this property suitable for displaying in the explorer/experimenter gui

---


### getKValue

```
public int getKValue()
```

:   Get the value of K.

    :   **Returns:**: Value of K.

---


### setKValue

```
public void setKValue(int k)
```

:   Set the value of K.

    :   **Parameters:**: `k` - Value to assign to K.

---


### seedTipText

```
public java.lang.String seedTipText()
```

:   Returns the tip text for this property

    :   **Returns:**: tip text for this property suitable for displaying in the explorer/experimenter gui

---


### setSeed

```
public void setSeed(int seed)
```

:   Set the seed for random number generation.

    :   **Specified by:**: `setSeed` in interface `Randomizable`
    :   **Parameters:**: `seed` - the seed

---


### getSeed

```
public int getSeed()
```

:   Gets the seed for the random number generations

    :   **Specified by:**: `getSeed` in interface `Randomizable`
    :   **Returns:**: the seed for the random number generation

---


### maxDepthTipText

```
public java.lang.String maxDepthTipText()
```

:   Returns the tip text for this property

    :   **Returns:**: tip text for this property suitable for displaying in the explorer/experimenter gui

---


### getMaxDepth

```
public int getMaxDepth()
```

:   Get the maximum depth of trh tree, 0 for unlimited.

    :   **Returns:**: the maximum depth.

---


### numFoldsTipText

```
public java.lang.String numFoldsTipText()
```

:   Returns the tip text for this property

    :   **Returns:**: tip text for this property suitable for displaying in the explorer/experimenter gui

---


### getNumFolds

```
public int getNumFolds()
```

:   Get the value of NumFolds.

    :   **Returns:**: Value of NumFolds.

---


### setNumFolds

```
public void setNumFolds(int newNumFolds)
```

:   Set the value of NumFolds.

    :   **Parameters:**: `newNumFolds` - Value to assign to NumFolds.

---


### allowUnclassifiedInstancesTipText

```
public java.lang.String allowUnclassifiedInstancesTipText()
```

:   Returns the tip text for this property

    :   **Returns:**: tip text for this property suitable for displaying in the explorer/experimenter gui

---


### getAllowUnclassifiedInstances

```
public boolean getAllowUnclassifiedInstances()
```

:   Get the value of NumFolds.

    :   **Returns:**: Value of NumFolds.

---


### setAllowUnclassifiedInstances

```
public void setAllowUnclassifiedInstances(boolean newAllowUnclassifiedInstances)
```

:   Set the value of AllowUnclassifiedInstances.

    :   **Parameters:**: `newAllowUnclassifiedInstances` - Value to assign to AllowUnclassifiedInstances.

---


### setMaxDepth

```
public void setMaxDepth(int value)
```

:   Set the maximum depth of the tree, 0 for unlimited.

    :   **Parameters:**: `value` - the maximum depth.

---


### listOptions

```
public java.util.Enumeration listOptions()
```

:   Lists the command-line options for this classifier.

    :   **Specified by:**: `listOptions` in interface `OptionHandler` **Overrides:**: `listOptions` in class `Classifier`
    :   **Returns:**: an enumeration over all possible options

---


### getOptions

```
public java.lang.String[] getOptions()
```

:   Gets options from this classifier.

    :   **Specified by:**: `getOptions` in interface `OptionHandler` **Overrides:**: `getOptions` in class `Classifier`
    :   **Returns:**: the options for the current setup

---


### setOptions

```
public void setOptions(java.lang.String[] options)
                throws java.lang.Exception
```

:   Parses a given list of options.


    Valid options are:

    ```
     -K <number of attributes>
      Number of attributes to randomly investigate
      (<0 = int(log_2(#attributes)+1)).
    ```

    ```
     -M <minimum number of instances>
      Set minimum number of instances per leaf.
    ```

    ```
     -S <num>
      Seed for random number generator.
      (default 1)
    ```

    ```
     -depth <num>
      The maximum depth of the tree, 0 for unlimited.
      (default 0)
    ```

    ```
     -N <num>
      Number of folds for backfitting (default 0, no backfitting).
    ```

    ```
     -U
      Allow unclassified instances.
    ```

    ```
     -D
      If set, classifier is run in debug mode and
      may output additional info to the console
    ```

    :   **Specified by:**: `setOptions` in interface `OptionHandler` **Overrides:**: `setOptions` in class `Classifier`
    :   **Parameters:**: `options` - the list of options as an array of strings **Throws:**: `java.lang.Exception` - if an option is not supported

---


### getCapabilities

```
public Capabilities getCapabilities()
```

:   Returns default capabilities of the classifier.

    :   **Specified by:**: `getCapabilities` in interface `CapabilitiesHandler` **Overrides:**: `getCapabilities` in class `Classifier`
    :   **Returns:**: the capabilities of this classifier **See Also:**: `Capabilities`

---


### buildClassifier

```
public void buildClassifier(Instances data)
                     throws java.lang.Exception
```

:   Builds classifier.

    :   **Specified by:**: `buildClassifier` in class `Classifier`
    :   **Parameters:**: `data` - the data to train with **Throws:**: `java.lang.Exception` - if something goes wrong or the data doesn't fit

---


### backfitData

```
public void backfitData(Instances data)
                 throws java.lang.Exception
```

:   Backfits the given data into the tree.

    :   **Throws:**: `java.lang.Exception`

---


### distributionForInstance

```
public double[] distributionForInstance(Instance instance)
                                 throws java.lang.Exception
```

:   Computes class distribution of an instance using the decision tree.

    :   **Overrides:**: `distributionForInstance` in class `Classifier`
    :   **Parameters:**: `instance` - the instance to compute the distribution for **Returns:**: the computed class distribution **Throws:**: `java.lang.Exception` - if computation fails

---


### toGraph

```
public java.lang.String toGraph()
```

:   Outputs the decision tree as a graph

    :   **Returns:**: the tree as a graph

---


### toGraph

```
public int toGraph(java.lang.StringBuffer text,
                   int num)
            throws java.lang.Exception
```

:   Outputs one node for graph.

    :   **Parameters:**: `text` - the buffer to append the output to: `num` - unique node id **Returns:**: the next node id **Throws:**: `java.lang.Exception` - if generation fails

---


### toString

```
public java.lang.String toString()
```

:   Outputs the decision tree.

    :   **Overrides:**: `toString` in class `java.lang.Object`
    :   **Returns:**: a string representation of the classifier

---


### numNodes

```
public int numNodes()
```

:   Computes size of the tree.

    :   **Returns:**: the number of nodes

---


### getRevision

```
public java.lang.String getRevision()
```

:   Returns the revision string.

    :   **Specified by:**: `getRevision` in interface `RevisionHandler` **Overrides:**: `getRevision` in class `Classifier`
    :   **Returns:**: the revision

---


### main

```
public static void main(java.lang.String[] argv)
```

:   Main method for this class.

    :   **Parameters:**: `argv` - the commandline parameters

---


### graph

```
public java.lang.String graph()
                       throws java.lang.Exception
```

:   Returns graph describing the tree.

    :   **Specified by:**: `graph` in interface `Drawable`
    :   **Returns:**: the graph describing the tree **Throws:**: `java.lang.Exception` - if graph can't be computed

---


### graphType

```
public int graphType()
```

:   Returns the type of graph this classifier represents.

    :   **Specified by:**: `graphType` in interface `Drawable`
    :   **Returns:**: Drawable.TREE


---


|  |  |  |
| --- | --- | --- |
| **Overview** \| **Package** \| **Class** \| **Tree** \| **Deprecated** \| **Index** \| **Help** \| **Weka's home** | |  |
| **PREV CLASS**   **NEXT CLASS** | **FRAMES**    **NO FRAMES**     **All Classes** |  |
| SUMMARY: NESTED \| FIELD \| CONSTR \| METHOD | DETAIL: FIELD \| CONSTR \| METHOD |  |


---
